# Comparative evaluation of image‐guided radiation therapy (IGRT)‐based dose calculation accuracy using cone‐beam, megavoltage, and kilovoltage CT modalities

**DOI:** 10.1002/acm2.70379

**Published:** 2025-11-21

**Authors:** Sirawit Saobai, Anirut Watcharawipha, Somsak Wanwilairat, Warit Thongsuk, Wannapha Nobnop

**Affiliations:** ^1^ Medical Physics Program Department of Radiology Faculty of Medicine ChiangMai University Chiang Mai Thailand; ^2^ Division of Radiation Oncology Department of Radiology Faculty of Medicine ChiangMai University Chiang Mai Thailand

**Keywords:** cone‐beam CT (CBCT), dose re‐calculation, Hounsfield unit‐to‐density calibration (HU‐D), IG‐kVCT, image‐guided radiation therapy (IGRT), MVCT

## Abstract

**Purpose:**

This study evaluates the dosimetric accuracy of three image‐guided radiotherapy (IGRT) imaging modalities, cone‐beam computed tomography (CBCT), megavoltage computed tomography (MVCT), and image‐guided kilovoltage computed tomography (IG‐kVCT), using modality‐specific HU‐to‐density (HU‐D) calibrations. Dose calculations from IGRT images were compared to computed tomography simulation (CT‐sim) references in phantoms and validated against measurements from head and neck and prostate patient plans to assess the feasibility of each modality for precise dose calculation in adaptive radiotherapy (ART).

**Methods:**

Two phantoms, the Tomo phantom HE and CIRS Thorax phantom, were used for HU‐to‐density (HU‐D) calibration. IGRT images were acquired using Elekta Synergy XVI (CBCT) and Radixact X9 (MVCT and IG‐kVCT), and calibration curves were generated for each modality. Dose distributions calculated from IGRT images were then compared with those from CT‐sim in phantom studies. For measurement‐based evaluation, 10 patient plans (head and neck and prostate cases) were delivered to a phantom and measured using the ArcCHECK system, and recalculated doses on CT‐sim and IGRT images were compared to the measured doses. Gamma analysis was performed to assess dosimetric accuracy.

**Results:**

IG‐kVCT showed the closest agreement with CT‐sim, achieving gamma passing rates (GPR) of 99.8% ± 0.3% for 3%/3 mm and 98.5% ± 0.7% for 3%/2 mm criteria, with dose differences below 1%. CBCT and MVCT demonstrated slightly lower accuracy, with GPRs of 97.2% ± 1.1% and 96.5% ± 1.3% for 3%/3 mm, respectively, and dose differences up to 2%. Similar trends were observed when compared to measured doses. All IGRT modalities showed clinically acceptable agreement, and no statistically significant differences were found between CT‐sim and any IGRT modality.

**Conclusion:**

All three IGRT modalities demonstrated clinically acceptable accuracy for adaptive dose calculation with modality‐specific HU‐D calibration curves.

## 1 INTRODUCTION

Image‐guided radiotherapy (IGRT) plays an important role in modern radiation therapy by helping to accurately locate and verify the position of the tumor before each treatment. In addition to improving setup accuracy, IGRT provides 3D imaging that can also be used for dose calculation and treatment adaptation. With imaging technologies such as cone‐beam computed tomography (CBCT), image‐guided kilovoltage computed tomography (IG‐kVCT), and megavoltage computed tomography (MVCT), clinicians can monitor changes in patient anatomy and adjust the treatment plan when needed. In some systems, daily imaging is part of adaptive radiotherapy (ART), allowing real‐time updates to the treatment plan.^1^, ^2^

A key factor that affects the accuracy of dose calculation from IGRT images is the Hounsfield Unit‐to‐density (HU‐D) calibration curve. This curve links the Hounsfield Units from CT images with the electron density of tissues, which is important for correct dose calculations in treatment planning.^3^ However, HU values derived from IGRT modalities, such as CBCT, MVCT, and IG‐kVCT, can differ significantly from those obtained through standard CT simulation (CT‐sim) scans due to differences in energy, beam characteristics, and image acquisition. These differences can affect the HU values, as reported by several previous studies.^4^, ^5^, ^6^ Therefore, using an improper calibration curve on IGRT images can lead to errors in dose calculation. For example, Richter et al.^7^ found significant dose errors when CT‐sim calibration curves were applied to CBCT images. Similarly, MVCT and IG‐kVCT images acquired from tomotherapy systems require modality‐specific calibration curves to ensure accurate dose calculations. Langen et al.^8^ reported that using an inappropriate HU‐D table for MVCT image recalculation could result in dose calculation errors of up to 3.1%. For IG‐kVCT, such as ClearRT on the Radixact X9 system (Accuray, Sunnyvale, CA, USA), the vendor recommends performing HU‐D calibration for each image protocol variation when dose calculation is conducted on ClearRT images.^9^ Tegtmeier et al.^10^ demonstrated that using calibration curves matched to specific scan settings can improve accuracy for MVCT and ClearRT (IG‐kVCT) imaging, with errors of less than 2% and 1%, respectively, compared to planning CT‐sim. These findings highlight the need for calibration curves specific to each imaging method or protocol.

Interestingly, some studies have also reported that using the original CT‐sim HU‐D calibration curve can still provide adequate accuracy in certain cases. Lim et al.^11^ characterized novel iterative reconstructed CBCT images on L‐shaped linacs and showed that dose calculations based on the CT‐sim calibration table could achieve clinically acceptable accuracy for dose tracking and ART. This highlights that while modality‐specific calibration generally improves accuracy, reusing CT‐sim HU‐D curves may still be a practical option in some clinical workflows. The trade‐off between convenience and dosimetric accuracy should therefore be carefully considered depending on the imaging system, protocol, and clinical objective.

Although previous studies have investigated the dosimetric accuracy of CBCT, MVCT, and IG‐kVCT for image‐guided radiotherapy, most were limited to single‐platform analyses or phantom‐only validations without addressing workflow efficiency or clinical implementation.^7^, ^8^, ^10^ The present study expands on these findings by performing a cross‐platform evaluation using two independent treatment planning systems (Precision and Monaco) and by applying modality‐specific HU‐D calibration curves derived under consistent imaging conditions. This approach provides a more clinically realistic assessment of IGRT‐based dose recalculation accuracy and demonstrates the potential for improved adaptive workflows by using daily IGRT images without the need for repeat CT simulations.

Despite these challenges, the utilization of daily IGRT images for adaptive radiotherapy is increasingly being adopted in clinical practice. Proper HU‐D calibration remains a critical component to achieve accurate dose calculations. In clinics that utilize both C‐arm linear accelerators and ring‐based systems, which rely on different IGRT modalities, it is important to establish and verify calibration curves for each imaging type. This study aims to evaluate the accuracy of dose calculation using IGRT images from different systems, including CBCT from C‐arm linear accelerators Elekta Synergy XVI system (Elekta AB, Stockholm, Sweden), and MVCT and IG‐kVCT from ring‐based tomotherapy systems, Radixact X9 (Accuray, Sunnyvale, CA, USA). The dose results from HU‐D calibrated IGRT images were compared with those from CT‐sim plans to see how reliable IGRT‐based adaptive radiotherapy can be in clinical practice.

## 2 METHODS AND MATERIALS

### 2.1 Phantom plans

**FIGURE 1:**
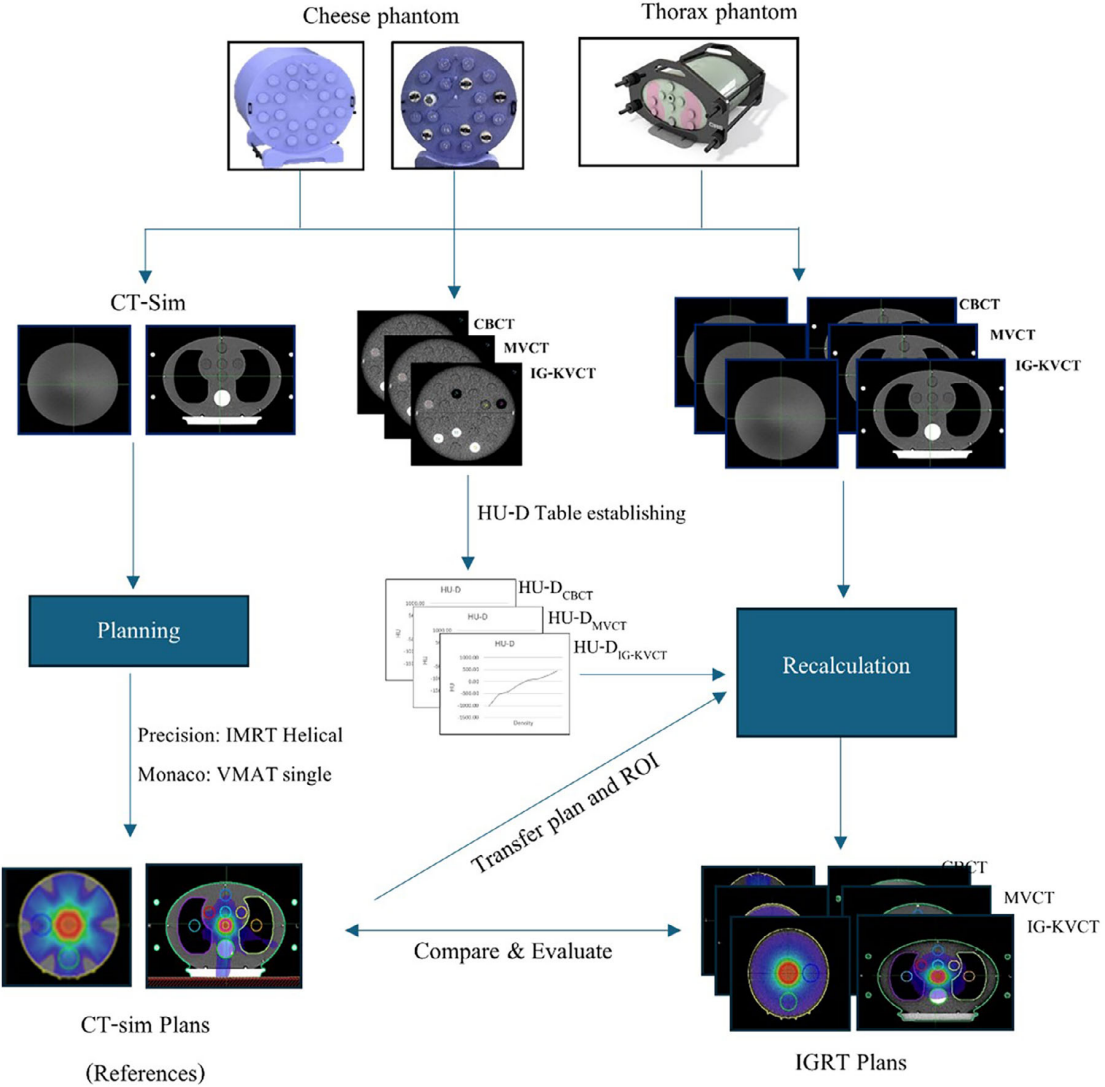
Workflow for generating HU‐D calibration tables and recalculating doses on IGRT images using cheese and thorax phantoms, followed by comparison with CT‐sim plans. CT‐sim, computed tomography simulation; HU‐D, HU‐to‐density; IGRT, image‐guided radiotherapy.

For the methodological workflow illustrated in Figure 1, the Cheese phantom (Tomo‐phantom HE Sun Nuclear Corporation, Melbourne, Florida, USA) was used in this study for both dose calculation and the establishment of HU‐D calibration, utilizing heterogeneity density plugs. This phantom featured 20 density insertion ports, all of which were filled with water‐equivalent density plugs to create a homogeneous model for initial dose calculations. To simulate a more realistic patient anatomy, the CIRS Model 002LFC Thorax phantom (CIRS Inc., Asbury Ave, Norfolk, Virginia 23513, USA) was also employed. This torso‐shaped phantom is composed of tissue‐equivalent materials and includes synthetic representations of organs such as lungs, bones, and soft tissue. Its design enabled the evaluation of how inhomogeneous regions influence dose calculations in IGRT.

#### 2.1.1 Image acquisition

CT‐sim imaging was performed using a SIEMENS Somatom Sensation scanner (Siemens Healthineers, Erlangen, Germany) for both the Cheese and Thorax phantoms. A thorax imaging protocol was applied, with parameters set at a 2 mm slice thickness, 120 kV, 185 mAs, and a 390 mm field of view (FOV). The CTDI_vol_ was 5.9 mGy for the Thorax phantom acquisition.

CBCT images were acquired using the Elekta Synergy XVI system (Elekta AB, Stockholm, Sweden) with the standard clinical thorax protocol. The CBCT imaging parameters included 120 kV, 264 mAs, a reconstructed slice thickness of 2 mm, an M20 collimator, and an F1 bowtie filter. The rotation time (60 s) and pitch corresponded to the default vendor settings. The CTDI was 3.8 mGy for both the Cheese and Thorax phantoms.

MVCT and IG‐kVCT imaging were performed using the Radixact X9 system (Accuray, Sunnyvale, CA, USA), with acquisition parameters tailored to the anatomical region and dose calculation grid size. For IG‐kVCT, the Cheese phantom was scanned using the ‘Head Small’ protocol (120 kVp, 240 mAs, 440 mm FOV, Fine mode, 1.4 mm slice interval, and 2.4 mm slice thickness). For the Thorax phantom, the ‘Thorax Medium’ protocol was applied (120 kVp, 375 mAs, 440 mm FOV, Fine mode, 1.8 mm slice interval, and 3.6 mm slice thickness).

In the Radixact X9 system, the imaging mode selection (e.g., Fine, Normal, or Coarse) determines the longitudinal beam width, couch translation speed, and the number of projection views per rotation. The Fine mode provides higher longitudinal resolution and improved soft‐tissue contrast by employing a smaller beam width and slower couch movement.^10^ For both protocols, the rotation time (30 s) and helical pitch (1.0) followed the vendor's default settings. The CTDI values were 0.9 and 1.3 cGy for the Cheese and Thorax phantoms, respectively.

The CT‐sim images were transferred to MIM Maestro software (MIM Software Inc., Cleveland, OH, USA) for organ contouring, which served as the basis for dose calculations, as illustrated in Figure 1. These images and the associated anatomical structures were used to generate treatment plans, perform dose recalculations, and compare IGRT‐based plans. Additionally, CBCT, MVCT, and IG‐kVCT images of both phantoms were imported into MIM. The structures delineated on the CT‐sim images were mapped onto the IGRT images using rigid registration.

### 2.2 Hounsfield Unit‐Density Tables Establishment

For each imaging modality, an HU‐D calibration curve was established using a direct calibration method. The Cheese phantom was scanned with seven different density insert plugs using CBCT, MVCT, and IG‐kVCT imaging. The inserts included air, LN‐300 (lung), LN‐450 (lung), true water, inner bone, CB2‐30%, CB2‐50%, and cortical bone. The acquired images were then imported into MIM Maestro software. Regions of interest (ROIs) with an 8‐mm diameter were placed on each plug to obtain mean HU values, which were subsequently correlated with the known physical densities (g/cm^3^) and relative electron densities. To ensure accuracy across different imaging systems, separate HU‐D calibration curves were generated for IG‐kVCT, MVCT, and CBCT modalities. Each calibration accounted for the specific x‐ray energy spectra, reconstruction algorithm, and image processing characteristics unique to each system, thereby minimizing potential discrepancies in HU‐D conversion that could affect dose calculation accuracy. As a result, modality‐specific HU‐D tables were generated for each imaging modality: HU‐D_CBCT_ for CBCT, HU‐D_MVCT_ for MVCT, and HU‐D_IG‐kVCT_ for IG‐kVCT.

#### 2.2.1 Treatment planning

To evaluate the dose calculation accuracy, treatment plan simulations were performed on CT‐sim images following the AAPM TG148 Tomophant plans^12^ and IAEA TECDOC 1583^13^ for target and ROI delineation on Cheese phantom and Thorax phantom, respectively. These data were exported to the Precision treatment planning system (TPS) version 3.3.1.3 (Accuray, Sunnyvale, CA, USA) and Monaco TPS version 6.1.3.0 (Elekta AB, Stockholm, Sweden) to create the reference CT‐sim plans. The Precision TPS utilized a helical tomotherapy (HT) technique, while Monaco TPS employed a single‐arc volumetric modulated arc therapy (VMAT) technique. Both plans prescribed a dose of 2 Gy in a single fraction to the center of the planning target volume (PTV), serving as the baseline reference plans.

The use of two distinct phantoms and treatment techniques was intentional to reflect both controlled and clinically realistic planning scenarios. The Cheese phantom, representing a homogeneous geometry, allowed assessment of HU‐D calibration accuracy and algorithmic consistency under idealized conditions. In contrast, the Thorax phantom provided a heterogeneous anatomy that simulated patient‐like dose heterogeneity, particularly relevant for evaluating lung or thoracic regions. The combination of HT and VMAT techniques further enabled cross‐validation of dose recalculations across two widely used planning approaches and imaging platforms, thereby ensuring broader clinical relevance.

For dose recalculation, the CBCT images of both phantoms, along with the mapped structures, were imported into Monaco TPS. Dose was recalculated using the reference CT‐sim plan and the HU‐D_CBCT_ calibration table. MVCT and IG‐kVCT images, along with the same anatomical structures, were imported into the Precision TPS for dose recalculation using the HU‐D_MVCT_ and HU‐D_IG‐kVCT_ tables, respectively.

#### 2.2.2 Dose distribution comparisons (calculation on CT‐sim vs. calculation on IGRT‐images)

To evaluate the recalculated plans, dose distributions from the CT‐sim images (used as the reference plan) were compared with those from the IGRT‐based plans using gamma analysis. Two sets of gamma criteria were applied: 3 mm distance‐to‐agreement (DTA) with 2% dose difference and 3 mm DTA with 3% dose difference. These analyses, including the 2D gamma analysis, were performed with the SNC Patient Sun Nuclear Software version 8.5.1.9 (Sun Nuclear Corporation, Melbourne, FL, USA). Additionally, the mean percentage dose difference of the PTV was evaluated to further assess the agreement between the CT‐sim and IGRT‐based treatment plans.

### 2.3 Patient plans

#### 2.3.1 Image acquisition

To evaluate the accuracy of dose calculations in radiotherapy treatment plans based on IGRT images, the New Model 1220 ArcCHECK (Sun Nuclear Corporation, Melbourne, Florida, USA), a cylindrical phantom incorporating a helical diode array comprising 1386 detectors with a spatial resolution of 0.5 cm, was utilized. IGRT ArcCHECK images for all three modalities were acquired using the same imaging parameters as those employed for the Cheese and Thorax phantoms. For this process, verification plans were generated by mapping actual clinical treatment plans (head and neck and prostate) onto the ArcCHECK phantom following a standard patient‐specific quality assurance (PSQA) workflow. The dose distributions were recalculated on the ArcCHECK images using the same beam parameters and optimization settings as those applied in the original patient plans. The acquired images were then imported into the Precision and Monaco treatment planning systems to generate verification plans for dose evaluation.

#### 2.3.2 Treatment planning based on CT‐sim images

Radiotherapy treatment plans of five head and neck cancer patients and five prostate cancer patients, planned using VMAT and HT techniques, respectively, were used to create verification plans on the CT‐sim images of the ArcCHECK phantom. The Monaco TPS was employed for VMAT plans, while the Precision TPS was used for HT plans. Dose calculations were performed using HU‐D derived from the CT‐sim images. These plans served as reference treatment plans for PSQA process.

#### 2.3.3 Treatment planning based on IGRT images

The same head and neck and prostate cancer treatment plans, originally created on CT‐sim ArcCHECK images, were recalculated on IGRT‐based images using the corresponding HU‐D tables: HU‐D_CBCT_, HU‐D_IG‐kVCT_, and HU‐D_MVCT_. Dose recalculations were performed using the Monaco TPS for CBCT images and the Precision TPS for IG‐kVCT and MVCT images to evaluate dose calculation accuracy.

#### 2.3.4 Dose distribution comparisons (calculation vs. measurement)

Dose verification plans created on the ArcCHECK phantom from the CT‐sim image were delivered in the treatment room, and the 2D gamma passing rates (GPR) were recorded using 3%/3 mm and 3%/2 mm criteria. The comparison between calculated dose distributions from each IGRT image modality and the delivered dose was analyzed using GPR with the SNC Patient software.

## 3 RESULTS

The findings of this study are presented across multiple aspects, including HU‐D calibration, dose distribution comparisons across imaging modalities, and gamma analysis of treatment plan agreement.

**FIGURE 2:**
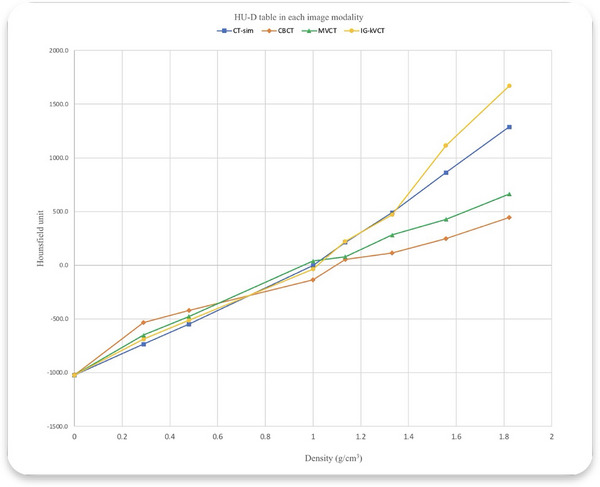
Correlation plot of HU values for HU‐D tables from each modality. HU‐D, HU‐to‐density.

The HU‐D calibration revealed distinct variations in HU values among the imaging modalities. As summarized in Table 1, the mean HU values were measured and correlated with the physical and relative electron densities of each inserted material. CT‐sim images exhibited HU values ranging from –1024 to 1288.6. Among the IGRT modalities, IG‐kVCT demonstrated the highest correlation with CT‐sim (*R* ^2^ = 0.987), while MVCT (*R* ^2^ = 0.964) and CBCT (*R* ^2^ = 0.934) showed lower agreement as illustrated in Figure 2.

**TABLE 1:** Relationship between Hounsfield Unit (HU), physical density, and relative electron density in each imaging modality.

	Density		HU							
Insert density plugs material	RED	Physical density (g/cm^3^)	CT‐sim		CBCT		MVCT		IG‐kVCT	
Air	0.0	0.0	−1024.0	0.0	−1024.0	0.0	−1022.3	2.9	−1024.0	0.0
LN‐300 (lung)	0.277	0.29	−735.0	5.8	−533.9	0.3	−651.2	0.4	−689.5	0.7
LN‐450 (lung)	4.63	0.48	−548.5	6.3	−420.5	0.6	−476.1	0.1	−513.9	1.2
True water	1.0	1.0	−3.9	0.9	−136.3	1.0	38.3	0.9	−36.8	1.0
Inner bone	1.092	1.135	213.0	2.3	53.9	0.7	79.6	0.6	221.1	1.1
CB2–30%	1.267	1.331	489.6	12.3	115.0	1.8	282.3	2.0	470.6	0.9
CB2–50%	1.46	1.557	861.5	6.0	248.6	1.7	427.3	1.2	1113.0	12.7
Cortical bone	1.687	1.822	1288.6	8.8	445.4	4.1	663.1	1.4	1670.1	0.2
–	–	–	Mean	SD	Mean	SD	Mean	SD	Mean	SD

Abbreviations: CBCT, cone‐beam computed tomography; CT‐sim, computed tomography simulation; GPR, gamma passing rates; HT, helical tomotherapy; IG‐kVCT, image‐guided kilovoltage computed tomography; MVCT, megavoltage computed tomography; RMS, root mean square; SD, standard deviation; VMAT, volumetric modulated arc therapy.

### 3.1 Phantom plans

**FIGURE 3:**
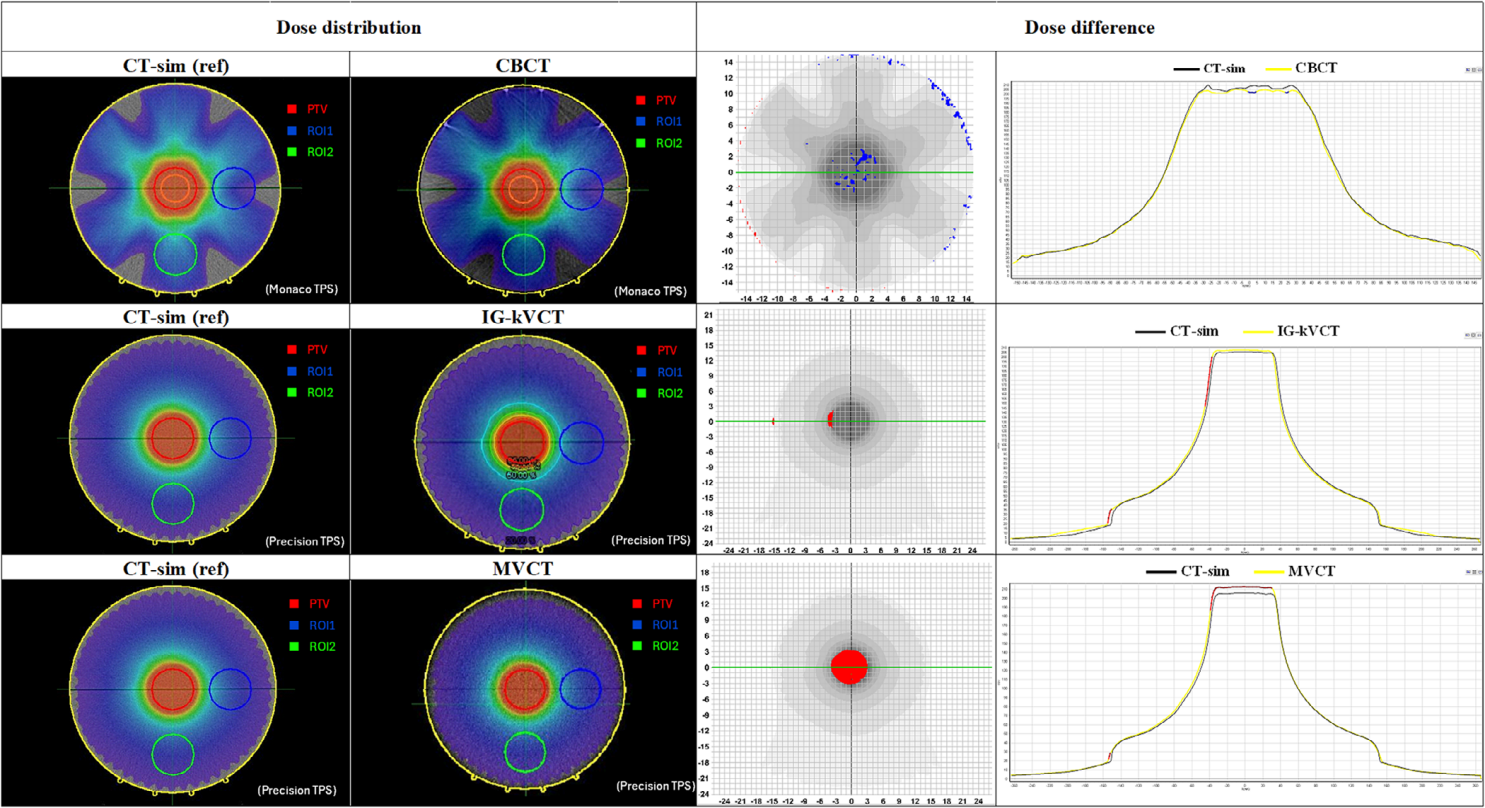
Example of dose distributions from CT and IGRT for each modality on the Cheese phantom. CT‐sim, computed tomography simulation; IGRT, image‐guided radiotherapy.

**FIGURE 4:**
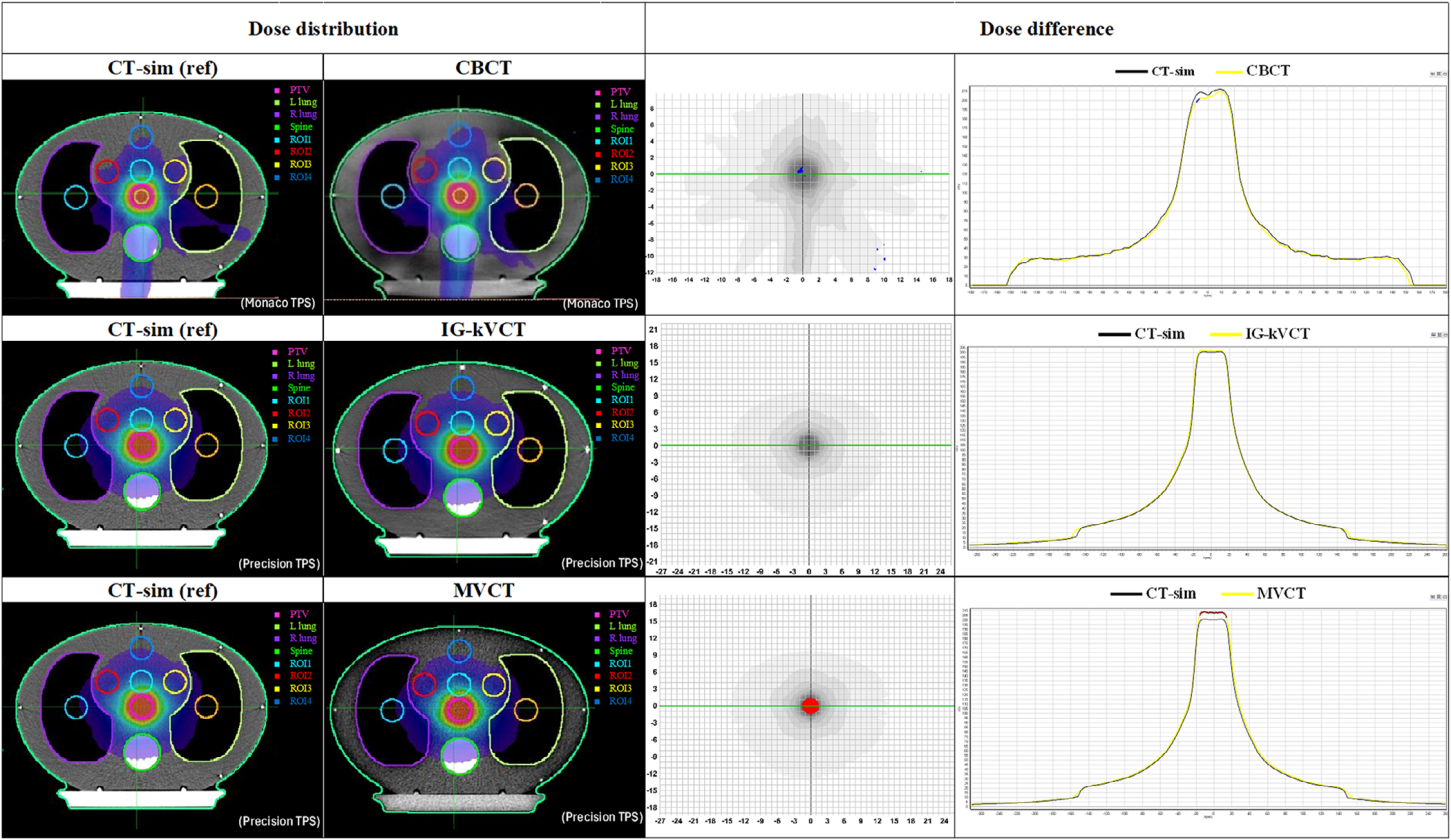
Example of dose distributions from CT and IGRT for each modality on the Thorax phantom. CT‐sim, computed tomography simulation; IGRT, image‐guided radiotherapy.

When comparing dose distributions, CBCT‐based calculations demonstrated generally minor deviations from the CT‐sim reference plans. Figures 3 and 4 illustrate the dose differences for both the Cheese and Thorax phantoms, with gamma analysis highlighting regions exceeding the 3%/2 mm criteria. Underdosed regions in the CBCT‐based plan are shown in blue, while red regions indicate overdosage. The dose profile demonstrates a lower dose level compared with the CT‐sim reference. For the PTV, the Cheese phantom showed a mean dose difference of −1.80% compared to CT‐sim, while the Thorax phantom demonstrated a slightly higher deviation of –1.94%. Importantly, the overall dose variation in the PTV remained within 2.00%, suggesting acceptable agreement between CBCT‐based and CT‐sim plans in homogenous media.

In contrast, dose distributions calculated from IG‐kVCT images tended to slightly overestimate the dose compared to CT‐sim in both phantoms. The red regions indicate areas of overdosage, and the dose profiles demonstrate a higher dose level compared with the CT‐sim reference, as shown in the last columns of Figures 3 and 4. The corresponding mean PTV dose difference was 0.98% for the Cheese phantom, while the Thorax phantom exhibited a mean difference of 0.97%. These results suggest that IG‐kVCT may slightly overpredict the delivered dose.

**FIGURE 5:**
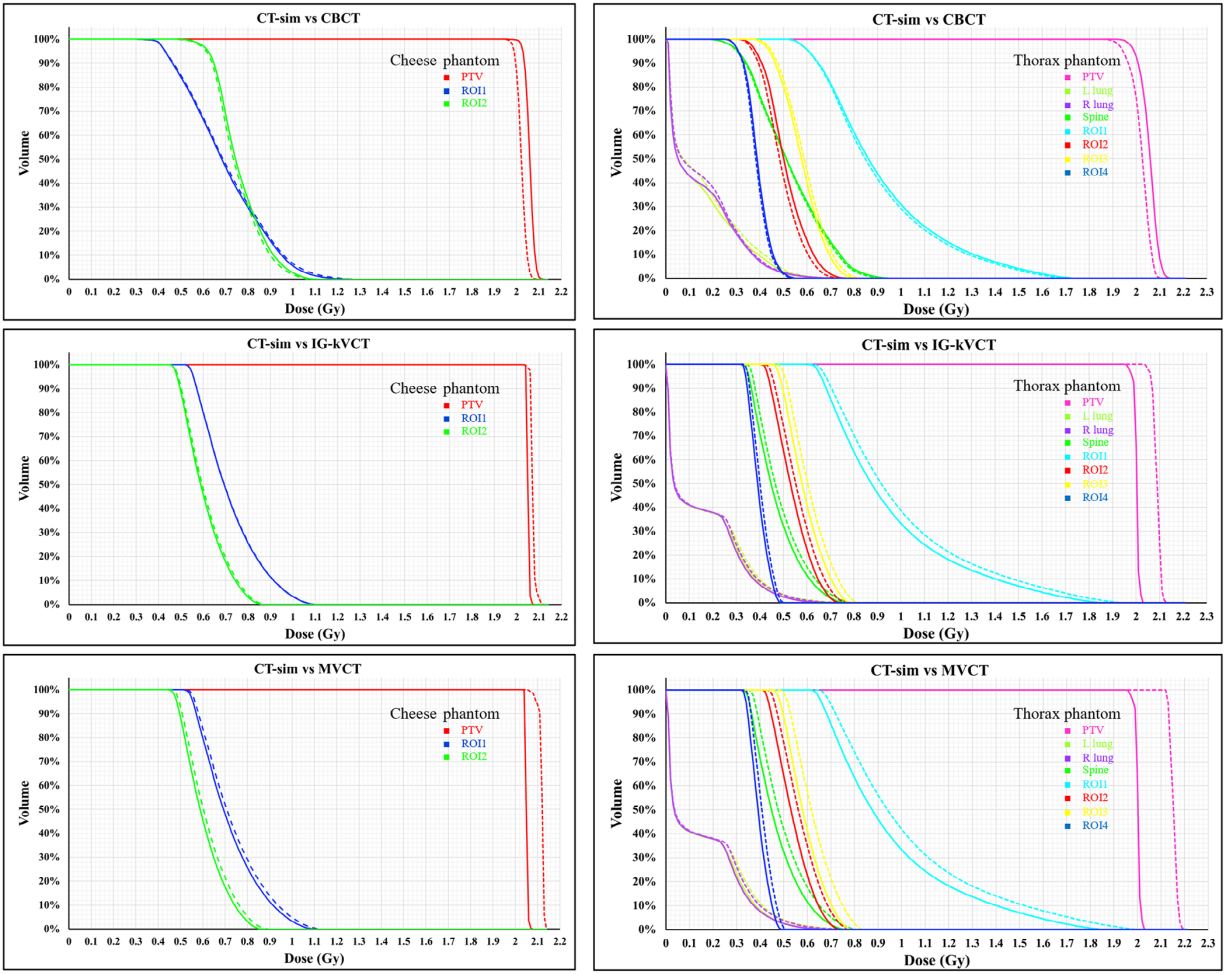
Comparison of dose–volume histograms (DVHs) between dose calculations on CT simulation (solid lines) and IGRT modalities (dashed lines) in Cheese and Thorax phantoms. CT‐sim, computed tomography simulation; IGRT, image‐guided radiotherapy.

The highest dose discrepancies were observed in the MVCT‐based plans. As shown in the last row of Figures 3 and 4, the MVCT images exhibited the greatest spatial variations in dose distribution compared with the CT‐sim reference. Similar to IG‐kVCT, the MVCT‐based dose calculations tended to slightly overestimate the delivered dose; however, the magnitude of these deviations was greater. These findings are consistent with those reported by Tegtmeier et al.,^10^ who also demonstrated that both IG‐kVCT‐based and MVCT‐based dose calculations slightly overestimated the delivered dose in Thorax phantom studies. This agreement further supports the validity of the present study's results and indicates that such overestimations may be inherent to image‐based dose recalculation processes in IGRT systems. Moreover, these results were consistent with the dose–volume histogram (DVH) comparisons of the PTV and ROI between CT‐sim and each IGRT modality in the Cheese and Thorax phantoms, as shown in Figure 5. The DVH analysis demonstrated small deviations for IG‐kVCT and larger deviations for MVCT.

To quantify the level of agreement between CT‐sim and IGRT‐based dose distributions, gamma analysis was performed using two commonly applied criteria: 3%/3 mm and 3%/2 mm. Table 2 summarizes the GPR. Across all IGRT modalities, GPR exceeded 95%, indicating generally good agreement with the CT‐sim reference. IG‐kVCT achieved the highest conformity, with a 100% GPR under the 3%/3 mm criterion for the Cheese phantom, and 99.7%–100% under the stricter 3%/2 mm criterion for both phantoms. In comparison, MVCT showed the lowest GPR. For the Cheese phantom, the GPRs were 96.9% and 96.7% for the 3%/3 mm and 3%/2 mm criteria, respectively. Slightly better performance was observed in the Thorax phantom, with GPRs of 98.8% and 98.7% for the same criteria.

**TABLE 2:** Comparison of mean dose difference and gamma passing rates of the IGRT plan compared to CT‐sim.

	CBCT			MVCT			IG‐kVCT		
Phantom	∆Dmean (%)	3%/3 mm	3%/2 mm	∆Dmean (%)	3%/3 mm	3%/2 mm	∆Dmean (%)	3%/3 mm	3%/2 mm
Cheese	−1.80	99.6%	98.9%	4.39	96.9%	96.7%	0.98	100%	99.7%
Thorax	−1.94	100%	99.9%	3.86	98.8%	98.7%	0.97	100%	100%

Abbreviations: CBCT, cone‐beam computed tomography; CT‐sim, computed tomography simulation; GPR, gamma passing rates; IG‐kVCT, image‐guided kilovoltage computed tomography; IGRT, image‐guided radiotherapy; MVCT, megavoltage computed tomography.

### 3.2 Patient plans

To assess the accuracy of dose calculation compared to measurements using IGRT images, treatment plans of five head and neck cancer patients and five prostate cancer patients were recalculated using images from three IGRT modalities: IG‐kVCT, MVCT, and CBCT. The recalculated plans, based on VMAT and HT techniques, were evaluated using the ArcCHECK phantom. Dosimetric accuracy was analyzed using GPR with criteria of 3%/3 mm and 3%/2 mm, as shown in Table 3.

**TABLE 3:** Comparison of gamma passing rates between calculated dose distributions from CT simulation and image‐guided modalities (CBCT, IG‐KVCT, MVCT) versus measured dose distributions for VMAT and HT plans.

Metric		Head and Neck		Prostate	
		3%/3 mm	3%/2 mm	3%/3 mm	3%/2 mm
**VMAT (Monaco): CBCT**					
CT‐sim vs. measurement	GPR (mean ± SD)	98.1 ± 1.4	95.2 ± 3.3	97.8 ± 1.1	94.1 ± 1.0
CBCT vs. measurement	GPR (mean ± SD)	98.3 ± 1.5	95.3 ± 3.5	98.3 ± 0.8	96.1 ± 1.7
% Difference CT‐sim vs. CBCT	RMS (%)	1.49			
	*p*‐value	0.43			
**HT (Precision): IG‐kVCT**					
CT‐sim vs. measurement	GPR (mean ± SD)	99.7 ± 0.4	99.3 ± 0.6	99.9 ± 0.2	99.5 ± 0.4
IG‐kVCT vs. measurement	GPR (mean ± SD)	99.8 ± 0.2	99.6 ± 0.6	99.4 ± 1.2	98.9 ± 1.8
% Difference CT‐sim vs. IG‐kVCT	RMS (%)	1.07			
	*p*‐value	0.53			
**HT (Precision): MVCT**					
CT‐sim vs. measurement	GPR (mean ± SD)	99.7 ± 0.4	99.3 ± 0.6	99.9 ± 0.2	99.5 ± 0.4
MVCT vs. measurement	GPR (mean ± SD)	99.6 ± 0.4	96.1 ± 4.2	100 ± 0.1	99.4 ± 0.6
% Difference CT‐sim vs. MVCT	RMS (%)	2.39			
	*p*‐value	0.16			

Abbreviations: CBCT, cone‐beam computed tomography; CT‐sim, computed tomography simulation; GPR, gamma passing rates; HT, helical tomotherapy; IG‐kVCT, image‐guided kilovoltage computed tomography; MVCT, megavoltage computed tomography; RMS, root mean square; SD, standard deviation; VMAT, volumetric modulated arc therapy.

For head and neck cancer cases, IG‐kVCT‐based verification plans demonstrated the highest agreement with the reference plans, yielding a mean GPR of 99.8 ± 0.2% for the 3%/3 mm criterion and 99.6 ± 0.6% for the 3%/2 mm criterion. MVCT‐based plans showed comparable results, with a GPR of 99.6 ± 0.4% for 3%/3 mm; however, the GPR decreased to 96.1 ± 4.2% for 3%/2 mm. CBCT‐based plans showed GPRs of 98.3 ± 1.5% and 95.3 ± 3.5% for the 3%/3 mm and 3%/2 mm criteria, respectively.

For prostate cancer cases, a similar trend was observed. IG‐kVCT‐based verification plans again showed the highest agreement, with a mean GPR of 99.4 ± 1.2% for the 3%/3 mm criterion and 98.9 ± 1.8% for 3%/2 mm. MVCT‐based plans achieved a perfect GPR of 100 ± 0.1% for 3%/3 mm, but the GPR dropped to 99.4 ± 0.6% for 3%/2 mm. CBCT‐based plans showed GPRs of 98.3 ± 0.8% and 96.1 ± 1.7% for 3%/3 mm and 3%/2 mm, respectively.

Across both head and neck and prostate cases, IG‐kVCT demonstrated the lowest root mean square (RMS) difference of 1.07% when compared with the CT‐sim GPR (*p* = 0.53), followed by CBCT with an RMS of 1.49% (*p* = 0.43). MVCT showed a higher RMS of 2.39% (*p* = 0.16). Nevertheless, all three IGRT modalities yielded acceptable GPRs, with no statistically significant differences compared to the CT‐sim reference.

These findings suggest that all three IGRT imaging modalities are clinically acceptable for adaptive dose verification. IG‐kVCT showed the most consistent performance across both treatment sites. Although CBCT exhibited slightly lower agreement compared to IG‐kVCT, particularly under stricter gamma criteria, it remained within acceptable clinical tolerance. The results highlight the need to carefully consider modality‐specific HU‐D calibration when implementing adaptive radiotherapy workflows to maintain dose calculation accuracy.

## 4 DISCUSSION

This study evaluated the feasibility and accuracy of IGRT‐based dose calculations using modality‐specific HU‐D calibration curves for CBCT, IG‐kVCT, and MVCT images. All modalities achieved clinically acceptable GPR, confirming the potential of IGRT images for secondary dose verification and integration into adaptive radiotherapy workflows.

Among the evaluated modalities, IG‐kVCT demonstrated the highest concordance with CT‐sim. The consistently superior performance of IG‐kVCT supported previous work by Yang et al.,^14^ who attributed its improved dosimetric reliability to better soft‐tissue contrast and reduced scatter compared with CBCT and MVCT. This suggested that IG‐kVCT was particularly advantageous in adaptive planning scenarios that required precise dose verification. CBCT‐based recalculations showed a minor trend toward underdosage, yet GPR remained above 98%. This finding, consistent with Souleyman et al.,^15^ highlighted the importance of modality‐specific HU‐D calibration and protocol optimization, which could effectively compensate for CBCT‐related limitations. The ability to achieve clinically acceptable accuracy despite inherent image quality challenges underscores the practicality of CBCT when tailored calibration is employed.

MVCT‐based dose recalculations exhibited the largest deviations among the three modalities in both phantoms. Although these deviations remained within clinically acceptable limits, the findings highlighted inherent limitations in MVCT‐based dose accuracy, even when tailored HU‐D calibration tables were applied. One possible explanation is the variability in the process of generating HU‐D calibration curves for MVCT images, particularly when the ROIs were drawn in areas with densities close to that of water. According to AAPM TG‐148,^12^ variations in HU values within water‐equivalent regions have the greatest impact on dose calculation accuracy and therefore require regular verification. Another potential factor is the finer dose grid size commonly employed in MVCT‐based planning. As reported by Kawashima et al.,^16^ smaller grid sizes can lead to slightly higher calculated doses due to increased voxel resolution and greater sensitivity to steep dose gradients.

Additional variability in MVCT dose calculations could have arisen from acquisition parameters such as slice interval and reconstruction algorithms. Velten et al.^17^ reported that the choice of slice interval and reconstruction method affected image quality, HU stability, and image noise. Similarly, Hoshida et al.^18^ suggested that these factors warranted further investigation to improve the utility of MVCT in quantitative planning.

Patient‐specific analyses further confirmed these modality‐specific trends. IG‐kVCT achieved the highest reliability across both head and neck and prostate cancer cases, whereas MVCT showed reduced performance under stricter gamma criteria, particularly for head and neck plans. CBCT demonstrated intermediate accuracy, but its use was constrained by limited scan length, which could have excluded parts of elongated target volumes. As Richter et al.^7^ emphasized, complete anatomical coverage was critical in adaptive workflows, and this limitation needed to be considered in clinical implementation. Importantly, no significant differences in GPR were observed between IGRT‐based and CT‐sim plans across VMAT and HT techniques, supporting the robustness of IGRT‐based recalculation even for complex planning strategies.

This limitation was particularly relevant for CBCT systems with restricted axial scan lengths. The Elekta Synergy XVI system used in this study provided a maximum scan range of approximately 26 cm.^19^ To mitigate the risk of incomplete anatomy and underestimation of dose, prostate cases with PTVs exceeding this scan length were excluded from the analysis. These considerations aligned with the findings of Richter et al.,^7^ who highlighted the importance of full anatomical coverage in adaptive radiotherapy workflows using CBCT images.

In addition to confirming previously reported trends, this study fills an important gap by systematically validating IGRT‐based dose recalculations across two distinct image‐guidance platforms, Elekta (CBCT) and Radixact (MVCT and IG‐kVCT), under standardized HU‐D calibration conditions. This dual‐system approach provides a comprehensive assessment of cross‐platform reproducibility using identical phantoms, imaging protocols, and dose analysis criteria. The inclusion of both phantom and clinical QA evaluations further strengthens the translational relevance of the findings, supporting the clinical integration of IGRT‐based adaptive dose verification in institutions employing multiple treatment systems.

From a clinical standpoint, the results of this study support the feasibility of integrating IGRT‐based dose recalculations into routine adaptive workflows. Specifically, IG‐kVCT showed the closest agreement with CT‐sim in both dose measurement and calculation, suggesting that it may serve as a practical surrogate for repeat CT‐sim in adaptive radiotherapy. By utilizing daily IGRT images for dose recalculation, the workflow can be simplified and treatment efficiency improved without compromising dosimetric accuracy. In contrast, although CBCT and MVCT achieved clinically acceptable accuracy, they may be less reliable in certain scenarios—such as large anatomical sites, regions with high tissue heterogeneity, or when limited scan length results in incomplete anatomical coverage. These findings provide guidance for selecting appropriate imaging modalities for adaptive dose verification based on clinical conditions and system capability.

In addition to its dosimetric accuracy, the clinical advantage of the proposed approach lies in the potential to streamline adaptive workflows by eliminating the need for repeat CT‐sim. By utilizing daily IGRT images, particularly IG‐kVCT and CBCT, for dose recalculation, the adaptive process can be completed using data already available from on‐board imaging. This not only reduces the time required for patient setup and re‐CT‐sim but also minimizes scheduling delays that often occur when patients must wait for re‐CT‐sim sessions. Furthermore, avoiding unnecessary repeat CT‐sim helps reduce the patient's cumulative imaging dose, thereby improving overall safety and workflow efficiency in adaptive radiotherapy.

Overall, these findings demonstrated that IGRT‐based dose recalculations were feasible and accurate for both phantom and clinical settings. IG‐kVCT appeared to offer the highest reliability, while CBCT and MVCT remained clinically usable with proper calibration and protocol refinement. From a broader perspective, this study contributed to the growing body of evidence supporting the role of modality‐specific IGRT images in adaptive radiotherapy. Continued optimization of CBCT calibration and MVCT acquisition protocols would further enhance the accuracy and clinical applicability of these imaging modalities in quantitative treatment planning.

## 5 CONCLUSION

This study demonstrated that IGRT‐based dose recalculations using modality‐specific HU‐D calibration curves can achieve high dosimetric accuracy, with GPR exceeding 95% across all modalities. IG‐kVCT showed the highest consistency, while CBCT and MVCT also provided acceptable results, though MVCT exhibited slightly larger dose deviations. These findings support the feasibility of using IGRT images for adaptive radiotherapy via modality‐specific HU‐D calibration.

## CONFLICT OF INTEREST STATEMENT

No conflicts of interest.
